# Diagnostic implications of digital breast tomosynthesis in symptomatic patients

**DOI:** 10.1186/bcr3782

**Published:** 2015-11-05

**Authors:** Sanjeeva Ramasundara, Lorraine Tucker, Matthew Wallis, Peter Britton, Penny Moyle, Kethryn Taylor, Ruchi Sinnatamby, Alan Freeman, Matthew Gaskarth, Fiona Gilbert

**Affiliations:** 1 Cambridge University Hospital, Cambridge, UK; 2 University of Cambridge, UK

## Introduction

The purpose of this study was to assess the diagnostic performance/utility of digital breast tomosynthesis (DBT) in symptomatic patients in a multidisciplinary clinical setting.

## Methods

The study was registered as a Cambridge University Hospital (CUH) service evaluation audit. Patients from the CUH symptomatic breast clinic from October 2014 to February 2015 were included in the study. Patients were included as clinic workflow permitted and DBT and full field digital mammography (FFDM) (SenoClaire, GE) were interpreted prospectively. Image quality of the DBT and 2D synthetic images were rated on a 5-point scale compared to FFDM. The imaging findings were graded on both FFDM and DBT as normal, benign, indeterminate, suspicious or malignant. Patients were clinically examined and additional ultrasound was carried out as appropriate. FFDM and DBT findings were correlated with the ultrasound findings and when performed to histopathology.

## Results

A total of 134 patients were included. Eighty-five per cent of the synthetic images were considered qualitatively similar or better than the FFDM images. Nineteen lesions were graded as indeterminate, 10 lesions were graded as suspicious and six lesions were graded as malignant on FFDM, whereas three lesions were graded as indeterminate, four lesions were graded as suspicious and 14 lesions were graded as malignant on DBT. Seventy-nine per cent of the indeterminate lesions on FFDM were downgraded accurately by DBT and 16 % were upgraded accurately by DBT. There was one false negative and three false positives with FFDM and DBT.

## Conclusion

DBT helps characterise indeterminate lesions more accurately compared with FFDM in the symptomatic setting.

